# Luteolin mitigates hippocampal damage in a rat model of streptozotocin-induced diabetes

**DOI:** 10.17305/bb.2025.12305

**Published:** 2025-05-05

**Authors:** Ömür Gülsüm Deniz, Hayriye Soytürk, Aydın Him, Dilek Sağir, Ebru Annaç

**Affiliations:** 1 Department of Histology and Embryology, Faculty of Medicine, Bolu Abant Izzet Baysal University, Bolu, Türkiye; 2 Department of Poultry Science and Technology, Faculty of Agriculture, Bolu Abant Izzet Baysal University, Bolu, Türkiye; 3 Department of Physiology, Faculty of Medicine, Bolu Abant Izzet Baysal University, Bolu, Türkiye; 4 Department of Occupational Health and Safety, Faculty of Health Sciences, Sinop University, Sinop, Türkiye; 5 Department of Histology and Embryology, Faculty of Medicine, Adıyaman University, Adıyaman, Türkiye

**Keywords:** Diabetes mellitus, luteolin, hippocampus, neurodegeneration, oxidative stress, endoplasmic reticulum stress

## Abstract

Diabetes mellitus (DM) is a chronic metabolic disorder that poses a serious threat to human health by causing long-term damage to various vital organs. It leads to insulin resistance and disrupts carbohydrate, fat, and protein metabolism. This study aimed to investigate the protective effects of luteolin (Lut) against diabetes-induced damage in the hippocampus of rats, using immunohistochemical, histopathological, biochemical, and molecular approaches. Lut [20 µg/kg, intraperitoneally (i.p.)] was administered to counteract hippocampal damage induced by diabetes, which was experimentally triggered using streptozotocin at a dose of 50 mg/kg (i.p.). The experiment lasted 28 days and included 48 rats divided into six groups of eight: Control, DM, citrate buffer (solvent), DM+Lut, Lut, and dimethyl sulfoxide (solvent). In the DMgroup, there was a decrease in Bcl-2 gene expression and an increase in the expression levels of Bax, caspase-3, cytochrome c, activating transcription factor-6, and inositol-requiring enzyme-1, compared to the DM+Lut group. Histological analysis revealed greater neuronal degeneration, neuroinflammation, and apoptosis in the DM group than in the DM+Lut group. Biochemical analysis also supported these findings, as indicated by increased oxidative stress index values. These results suggest that Lut mitigates the toxic effects of oxidative and endoplasmic reticulum stress, enhances antioxidant defenses, and supports hippocampal function. The findings demonstrate Lut’s potential to prevent diabetes-induced hippocampal damage. Consequently, further research is strongly recommended to explore Lut as a therapeutic agent for diabetic neurodegeneration.

## Introduction

Diabetes mellitus (DM) is a metabolic disease characterized by elevated blood glucose levels. Both hereditary and environmental factors contribute to its development, which can lead to a range of complications [1]. In DM, impaired insulin production or utilization prevents proper glucose metabolism, resulting in chronic hyperglycemia [2, 3]. This persistent elevation in blood glucose causes systemic dysfunction and chronic damage to blood vessels, nerves, the brain, and other organs and tissues [4]. Glucose is the primary energy source for the brain; thus, insulin-related disturbances in DM can negatively impact cognitive health [5]. Insulin receptors are abundantly found in neurons located in regions critical for memory and learning, such as the hippocampus and cerebral cortex [6]. Chronic hyperglycemia increases the production of reactive oxygen species (ROS), which in turn activate immune cells [5]. Elevated numbers of TUNEL-positive and caspase-3-positive cells have been observed in the brains of rats with streptozotocin (STZ)-induced diabetes [7]. In addition to mitochondrial dysfunction, endoplasmic reticulum (ER) dysfunction plays a key role in diabetes-related neurodegeneration. This disrupts glucose metabolism by increasing free radical production, contributing to central nervous system (CNS) degeneration [8]. Diabetic rats have shown nuclear and chromatin degeneration, as well as ER enlargement and degranulation [9]. ER dysfunction can trigger an unfolded protein response, resulting in ER stress. Hyperglycemia-induced ROS can lead to apoptosis in various diabetic rat tissues through the activation of ER stress pathways [10]. Given the chronic nature of DM and the numerous secondary complications it causes, the disease poses a significant global burden, both financially and in terms of healthcare resources. Moreover, the lifelong progression of the condition often has a negative psychological impact on patients [11]. These factors underscore the importance of exploring natural anti-diabetic compounds that may help alleviate diabetes-related complications. Luteolin (Lut), a flavonoid with a 3′,4′,5,7-tetrahydroxyflavone structure, is commonly found in plants used in traditional medicine to treat various disorders [12]. Research has demonstrated that Lut possesses antioxidant, anti-cancer, anti-diabetic, anti-inflammatory, and neuroprotective properties [12–15]. Its antioxidant capabilities are linked to its ability to scavenge reactive oxygen and nitrogen species, chelate transition metals involved in the Fenton reaction, inhibit pro-oxidant enzymes, and enhance the activity of antioxidant enzymes [12, 14]. Free Lut has been detected in both blood and brain tissue following peripheral administration, indicating its ability to cross the blood–brain barrier [16–18]. Additionally, Lut has been shown to improve memory and reduce neuroinflammation in diabetic rats by mitigating oxidative stress [19, 20]. Another study reported that Lut alleviated depression-like behaviors by inhibiting ATF4 expression, a key factor in ER stress [21]. Further research has shown that Lut reduces cognitive impairment in high-fat diet-induced diabetic mice by lowering mitochondrial ROS and preventing cytochrome c (Cyt c) release. These effects are thought to occur via modulation of the c-Jun N-terminal kinase (JNK) signaling pathway, thus protecting neural cells [22]. Diabetes-related symptoms and complications represent a serious health issue, particularly in developing countries. Investigating anti-diabetic agents that can mitigate these complications is therefore of considerable importance. In this context, the present study used Wistar albino rats with STZ-induced diabetes and performed biochemical, histopathological, immunohistochemical, and molecular analyses to evaluate the protective effects of Lut on the hippocampus. The study aims to provide a comprehensive assessment of Lut’s potential to exert multi-faceted protective effects—particularly through its antioxidant capacity to counteract oxidative and ER stress—thereby highlighting its therapeutic promise as an anti-diabetic and neuroprotective agent.

## Materials and methods

### Experimental design

All experimental procedures were conducted at the Bolu Abant Izzet Baysal University Experimental Animals Application and Research Center, following approval (no. 2022/09/A3 dated 05/07/2023) from the university’s local ethics committee. The well-being of the animals and ethical principles were prioritized throughout the entire experiment. All procedures complied with the U.K. Animals (Scientific Procedures) Act, 1986 and associated guidelines, the EU Directive 2010/63/EU for animal experiments, and the National Institutes of Health Guide for the Care and Use of Laboratory Animals (NIH Publication No. 8023, revised 1978). Animal cadaver hippocampi were used in this study. The research involved 48 adult female Wistar albino rats, aged 2–4 months and weighing 200–250 g, obtained from the Bolu Abant Izzet Baysal University Experimental Animals Research Center. The animals were housed under controlled environmental conditions: temperature (24 ± 2 ^∘^C), humidity (55% ± 15), and a 12-h light/dark cycle. At the start of the study, rats were randomly divided into six groups of eight animals each. A power analysis was conducted using Minitab version 18.0 to determine the appropriate sample size. The control (Cont) group (Cont, *n* ═ 8) received 1 mL/kg saline solution intraperitoneally (i.p.) for 28 days. The DM group (*n* ═ 8) received a single i.p. dose of 50 mg/kg STZ dissolved in 0.1 M citrate buffer (CB) (pH 4.0) [23–25]. After 72 h, fasting blood glucose levels were measured via blood collected from the tail vein. Rats with glucose levels exceeding 250 mg/dL were classified as diabetic. The CB group (*n* ═ 8) was administered a single i.p. dose of 0.1 M CB (pH 4.0). The DM+Lut group (*n* ═ 8) received 50 mg/kg STZ as described above, and 72 h later, rats with blood glucose levels above 250 mg/dL were identified as diabetic and then treated with daily i.p. injections of 20 µg/kg Lut (Sigma-Aldrich, St. Louis, MO, USA) [26] for 28 days. The Lut group (*n* ═ 8) received daily i.p. injections of 20 µg/kg Lut dissolved in dimethyl sulfoxide (DMSO) for 28 days. The DMSO group (*n* ═ 8) received daily i.p. injections of 1 mL/kg 0.5% DMSO for 28 days.

### Induction of experimental diabetes model and fasting blood glucose measurement

To establish an experimental diabetes model, a single i.p. injection of STZ 50 mg/kg, Cayman, 13104) dissolved in 0.1 M CB (pH 4.0) was administered following an overnight fast [21]. To prevent hypoglycemia during the initial 12–24 h post-injection, animals received a 5% glucose solution in tap water. Food was made available immediately after STZ administration [27]. Seventy-two hours later, fasting blood glucose levels were measured using blood samples collected via tail vein puncture after another overnight fast. Samples were applied to glucometer strips (Lifechek Smart, TD-4360), and three readings were obtained per animal. Animals with fasting blood glucose levels ≥250 mg/dL were classified as diabetic and included in subsequent studies [28]. Body weight and fasting blood glucose were recorded for all groups at both the beginning and end of the study.

### Obtaining tissues and blood samples

At the end of the 28-day experimental period, brain tissues were collected following the withdrawal of 3 mL of intracardiac blood under anesthesia with 90 mg/kg ketamine (Ketalar^®^, Pfizer, İstanbul) and 10 mg/kg xylazine (Citanest^®^, AstraZeneca, İstanbul). Total antioxidant status (TAS) and total oxidant status (TOS) values were then determined. The left hippocampus was isolated for molecular analyses, while the right hippocampus was used for histopathological and immunohistochemical examinations. For molecular analyses, the tissues were stored at −80 ^∘^C in 2 mL RNase/DNase-free Eppendorf tubes. For histopathological and immunohistochemical analyses, the tissues were fixed in 10% buffered neutral formalin for two weeks.

### Histologic analysis

#### Preparation of tissue samples

After the two-week fixation period, the right hippocampi were placed into labeled tissue tracking cassettes, submerged in running water overnight, and then removed from the formalin solution. The tissues were dehydrated through a graded ethanol series (70%, 80%, 96%, and 100%), cleared with xylene, and infiltrated with paraplast. They were then embedded in square L-irons containing molten paraffin on a flat surface. All groups were labeled and positioned in L-irons to prevent direct contact with the tissue, thus concluding the tissue tracking process. For light microscopy, 4 µm-thick sections were cut from the paraffin blocks using a rotary microtome (Leica RM2125RT) following systematic random sampling: 1/100 for immunohistochemical analyses and 1/50 for histopathological analyses. The sections were placed in a 42 ^∘^C water bath, then mounted on slides and incubated overnight at 58 ^∘^C to remove paraplast. Histopathological analysis was performed after staining with cresyl violet, using a microscope equipped with a Carl Zeiss Axiocam ERc5 digital camera. For immunohistochemical analysis, sections were stained with Caspase-3 and Glial Fibrillary Acidic Protein (GFAP).

### Immunohistochemical analysis

Hippocampal sections, 4 µm thick, were analyzed for apoptosis and astrocyte activation using the Avidin-Biotin Peroxidase Complex technique, following the standard protocol provided in the commercial kit. Active caspase-3 (Anti-Caspase-3 antibody, ab4051) served as the primary antibody to detect apoptosis, while a GFAP monoclonal antibody (Roche, clone EP672Y) was used as a marker of neuroinflammation. For immunohistochemical analysis of GFAP and caspase-3, sections were examined under a light microscope. Immunoreactive cells were identified as the criterion for positive staining, and data from all groups were subjected to semi-quantitative analysis. Staining specificity was confirmed using negative Cont sections processed without the primary antibody on the same tissues. For caspase-3 evaluation, six randomly selected areas (20 µm^2^ each) were examined at 40× magnification per section. Digital images were captured using a Nikon Eclipse 80i light microscope equipped with a camera. The number of caspase-3-positive cells in each field was divided by the total cell count. Each section was evaluated across six fields on average. The percentage of stained cells in each field was scored as follows: no staining (0), <25% (1), 25%–50% (2), and >50% (3). Staining intensity was graded as none (0), low (1), moderate (2), or strong (3) [29]. The immunostaining intensity distribution index (IIDI) was calculated using the formula: IIDI ═ % stained cells × staining intensity. For each section, the IIDI represents the mean value across all six fields. GFAP staining was evaluated by randomly selecting 10 fields per animal at 20× magnification using the same microscope setup. Five sections and 50 images were analyzed for each animal. In each field, 200 cells (GFAP-positive and -negative) were counted, and the GFAP-positive cell density was calculated using the formula described in [30].




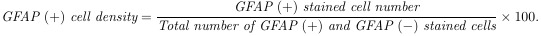




### Biochemical analysis

Following the completion of the experimental procedures, 3 mL of intracardiac blood was collected from anesthetized rats prior to sacrifice. The samples were placed into appropriate biochemistry tubes and centrifuged at 3000 rpm for 15 min at 4 ^∘^C. The resulting serum was stored at −80 ^∘^C until analysis. TAS and TOS levels were measured using biochemical methods with double antibody sandwich ELISA kits (Rat Serum TAS, Bioassay Technology Laboratory, Shanghai, China; catalogue no. E1710Ra, and Rat Serum TOS, Bioassay Technology Laboratory, Shanghai, China; catalogue no. E1512Ra).

### Molecular analysis

#### Q PCR method

JNK, inositol-requiring enzyme-1 (IRE1), and activating transcription factor-6 (ATF6)—three key biomarkers of ER stress—along with Bax, Caspase-3, Bcl-2, and Cyt c, which are associated with apoptosis, were analyzed in samples stored at −80 ^∘^C in 2 mL RNase/DNase-free Eppendorf tubes. GAPDH served as the housekeeping gene. Total RNA was extracted, followed by cDNA synthesis and quantitative real-time PCR (qRT-PCR) to assess changes in gene expression levels.

### RNA isolation

To isolate RNA from tissue samples, 50 mg of tissue was homogenized in 1 mL of TRIzol reagent. After incubation at room temperature for 5 min, 200 µL of chloroform was added and the mixture was shaken vigorously for 15 s. The samples were then left at room temperature for 3 min before being centrifuged at 12,000 × g for 15 min at 4 ^∘^C. The upper, transparent aqueous phase was carefully transferred to a new tube, and 500 µL of 100% isopropanol was added. After a 10-min incubation at room temperature, the tubes were centrifuged again at 12,000 × g for 10 min at 4 ^∘^C. At this stage, a white RNA precipitate was visible at the bottom of the tube. The supernatant was removed cautiously to avoid disturbing the pellet. The RNA pellet was then washed with 1 mL of 75% ethanol and centrifuged at 7500 × g for 5 min at 4 ^∘^C. Finally, the RNA was dissolved in 20–50 µL of DEPC-treated ddH_2_O, and the concentration was measured.

### cDNA synthesis

In summary, 1 µg of RNA, 2 µL of oligo (dT), and DEPC-treated ddH_2_O were combined to a final volume of 8 µL and incubated at 70 ^∘^C for 5 min. Following this, 10 µL of 2X reaction buffer and 2 µL of reverse transcriptase enzyme were added, and the mixture was incubated at 42 ^∘^C for 5 min, then at 80 ^∘^C for 1 h. The resulting cDNA samples were stored at −20 ^∘^C.

### qRT-PCR

Primers with high specificity to the target gene regions were tested for the RT-PCR experiments (Table 1). An amplification program was used to design the oligonucleotides and evaluate their properties, including melting temperature (Tm) and potential for primer-dimer formation. Primers were chosen from exon-intron junction regions to minimize off-target binding to nonspecific genomic sites. Primer specificity was further verified using in silico PCR via the UCSC Genome Browser. For qRT-PCR, each reaction contained 1 µL of cDNA, 1 µL of primer mix (10 µM, forward and reverse), 10 µL of 2X SYBR Green, and 8 µL of ddH_2_O. The thermal cycling protocol was as follows: initial denaturation at 95 ^∘^C for 5 min; 40 cycles of 95 ^∘^C for 15 s, 60 ^∘^C for 30 s, and 72 ^∘^C for 30 s; followed by a final extension at 72 ^∘^C for 5 min.

**Table 1:** Details of primers used in the present study

**Primer name**	**Primers (5′-3′)**	**Tm ^∘^C**
IRE1_F	GTCCAGCTGCTTCGAGAATC	59
IRE1_R	GATGAAGCAAGGTGATGGC	59
JNK_F	TGCCTTGGACTCTACTGTGG	59
JNK_R	TGCAAAACGAGTGGAAGCAT	55
ATF6_F	AGAGAAGCCTGTCACTGGTC	59
ATF6_R	TAATCGACTGCTGCTTTGCC	57
Caspase 3_F	TTTTGGAACGAACGGACCTG	57
Caspase 3_R	TGTCTCAATACCGCAGTCCA	57
Bax-F	GAGAGGATGGCTGGGGAGAC	63
Bax-R	GGTGAGCGAGGCGGTGAGGACT	68
Bcl 2-F	ATGGGGTGAACTGGGGGTGGATTG	66
Bcl 2-R	TTTCATATTTGTTTGGGGCAGGTC	59
Cytochrome c-F	TGGACAGCCCCGATTTAAGT	57
Cytochrome c-R	TCAATAGGTTTGAGGCGACAC	58
GAPDH-F	ACCACCATGGAGAAGGCTGG	61
GAPDH-R	CTCAGTGATGCCCAGGATGC	61

IRE1: Inositol-requiring enzyme-1; ATF6: Activating transcription factor-6; JNK: c-Jun N-terminal kinase; Tm: Melting temperature; Caspase-3: Cysteinyl aspartate specific proteinase-3; Bax: Bcl-2-associated X protein; Bcl-2: B-cell lymphoma 2; Cyt c: Cytochrome c; GAPDH: Glyceraldehyde-3-phosphate dehydrogenase.

### Analysis of the qRT-PCR results

To account for differences among samples and potential pipetting inaccuracies in the assessment of relative gene expression, normalization was performed using the housekeeping gene GAPDH. The analysis was conducted using the method shown in the following equation: Control ΔCT ═ Control CT (Gene) − Control CT (GAPDH)

Sample ΔCT ═ Sample CT (Gene) − Sample CT (GAPDH) ΔΔCT ═ [(Sample ΔCT) Avg. − (Control ΔCT) Avg.] Target Gene mRNA Quantity ═ 2^−ΔΔCT^

### Ethical statement

This study was approved by the Bolu Abant Izzet Baysal University Animal Research Local Ethics Committee, under decision number 2022/09/A3, dated 05/07/2023. The well-being of the experimental animals and adherence to ethical principles were prioritized throughout the experiment. All experimental procedures were conducted in accordance with the U.K. Animals (Scientific Procedures) Act of 1986 and its associated guidelines, the EU Directive 2010/63/EU on the protection of animals used for scientific purposes, or the National Institutes of Health Guide for the Care and Use of Laboratory Animals (NIH Publication No. 8023, revised 1978).

### Statistical analysis

Statistical analyses were performed using SPSS version 21.0. The Shapiro–Wilk test was used to assess the normality of data distribution. Normally distributed continuous variables were analyzed with one-way ANOVA followed by Bonferroni post hoc tests. For non-normally distributed data, the Kruskal–Wallis test and Bonferroni-adjusted Mann–Whitney *U* tests were used to compare multiple groups. A *P* value of less than 0.05 was considered statistically significant.

**Figure 1.**
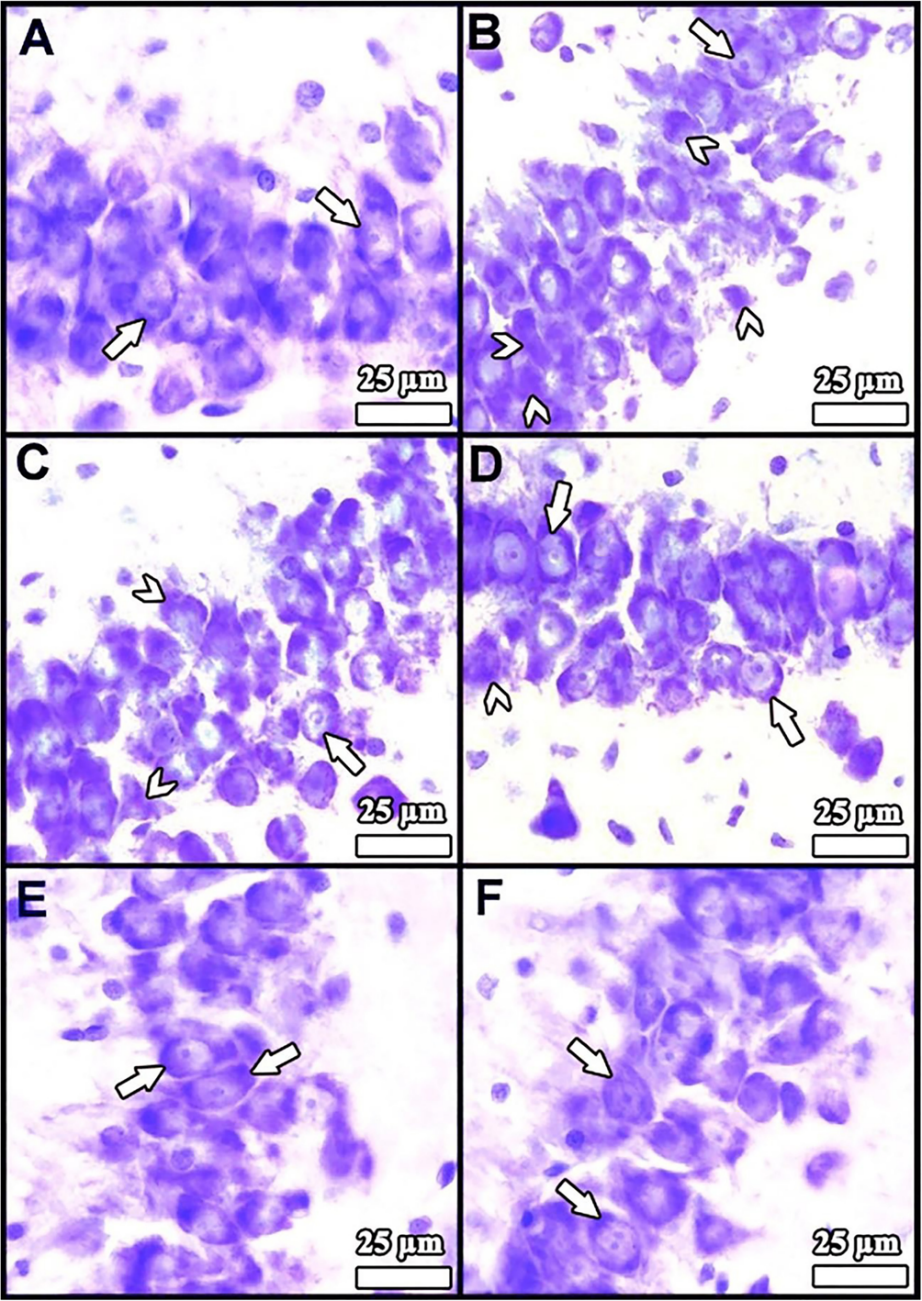
**Light microscopic sections from the hippocampus of rats from all groups (n ═ 8).** (A) Control group; (B) Diabetes mellitus group; (C) Diabetes mellitus + Luteolin group; (D) Luteolin group; (E) Citrate buffer group; (F) Dimethyl sulfoxide (solvent) group. (A, D, E, and F) revealed a normal appearance in the general structure and neurons. The large number of degenerated neurons was notable in (B). (C) demonstrated that side effects of diabetes were minimized by Lut. Arrows: Healthy neurons; Arrowheads: Degenerated neurons. Staining: Cresyl violet; magnification, ×1000.

## Results

### Histopathological results

Histopathological analysis of the hippocampi (Figure 1) revealed a normal structure and neuronal appearance in the Lut, DMSO, and CB groups. Light microscopy showed clearly defined borders of the pyramidal neuron perikarya. According to Sugimoto et al. [31], damaged neurons typically exhibit three features: irregular cellular outlines, and increased chromatin density in both the nucleus and cytoplasm. In the DM group, cell borders were indistinct, and a greater number of neurons showed narrow, dark-stained cytoplasm and possible degenerated cellular debris compared to the other groups. In contrast, the DM+Lut group exhibited more healthy neurons with clearly defined cell and nuclear borders than the DM group. Overall, histopathological examination indicated that Lut reduced DM-induced neuronal degeneration in the hippocampus.

### Immunohistochemical findings

#### GFAP staining findings

**Figure 2.**
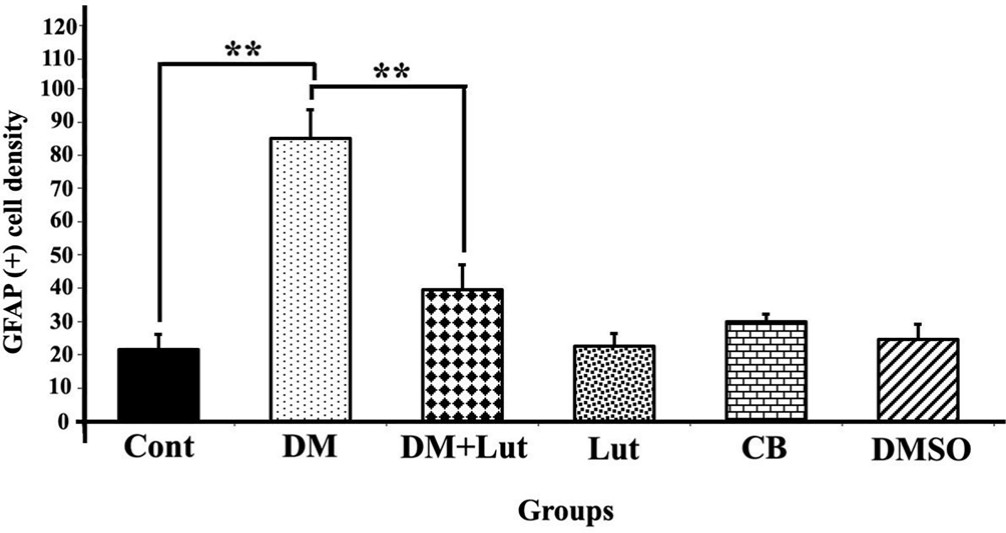
**The graph indicating GFAP (+) stained cell densities in the hippocampus regions from all groups (*n:* 8)**. Differences at the *P* < 0.01 level are indicated “**.” GFAP (+), a biomarker indicative of active astrocytes at locations of central nervous system injury, stained cell density was greater in the DM group compared to the other groups. Cont: Control; Lut: Luteolin; CB: Citrate buffer; DMSO: Dimethyl sulfoxide; DM: Diabetes mellitus; GFAP: Glial fibrillary acidic protein.

**Figure 3.**
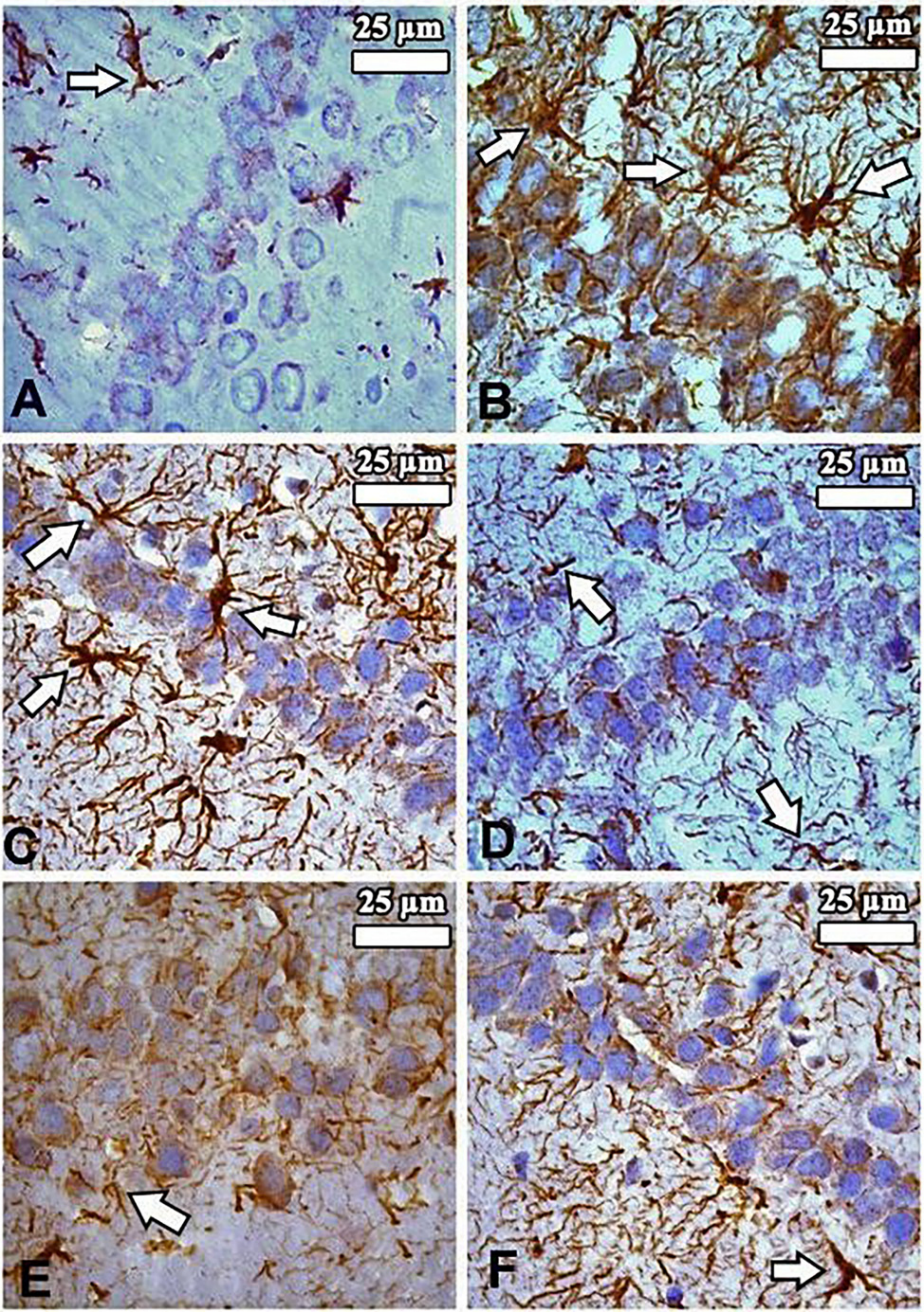
**Distribution of GFAP (+) stained cells in the hippocampus in the study groups (n: 8).** (A) Control group; (B) Diabetes mellitus group; (C) Diabetes mellitus + Luteolin group; (D) Luteolin group; (E) Citrate buffer group; (F) Dimethyl sulfoxide (solvent) group. Cytoplasmic extensions of GFAP (+) stained cells were greater in the (B) compared to the other groups. (C) indicated that Luteolin may minimize the inflammation that occurs due to diabetes. Arrows: GFAP (+) stained cells. Magnification: ×1000. GFAP: Glial fibrillary acidic protein.

The density of GFAP (+) stained cells in the hippocampi of rats was evaluated across all groups and subjected to statistical analysis (Figure 2). A statistically significant increase in GFAP (+) cell density was observed in the DM group compared to the others (*P* < 0.01). No significant differences were found among the Cont, Lut, CB, and DMSO groups (*P* > 0.05). Notably, the DM+Lut group showed a significantly lower GFAP (+) cell density than the DM group (*P* < 0.01). Additionally, GFAP (+) cells in the DM group exhibited more extensive cytoplasmic processes, which branched into multiple projections (Figure 3).

### Caspase-3 staining findings

**Figure 4.**
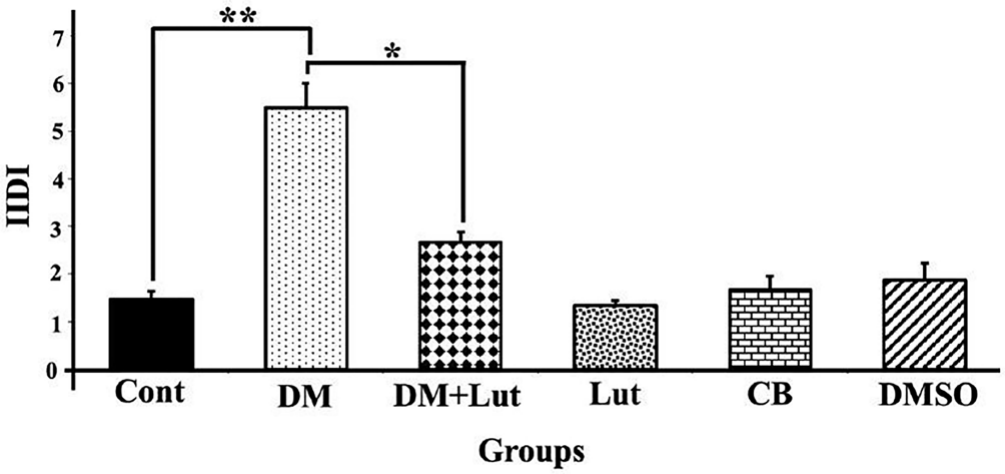
**IIDI values for caspase-3 immunoreactivity in the hippocampus region from all groups (*n*: 8).** Differences at the *P* < 0.05 level are indicated by “*” and those at the *P* < 0.01 level by “**”. Apoptosis in neurons of the DM group was significantly higher than in all the other groups. It is also observed that Lut has an antiapoptotic effect. Cont: Control; DM: Diabetes mellitus; Lut: Luteolin; CB: Citrate buffer; DMSO: Dimethyl sulfoxide.

**Figure 5.**
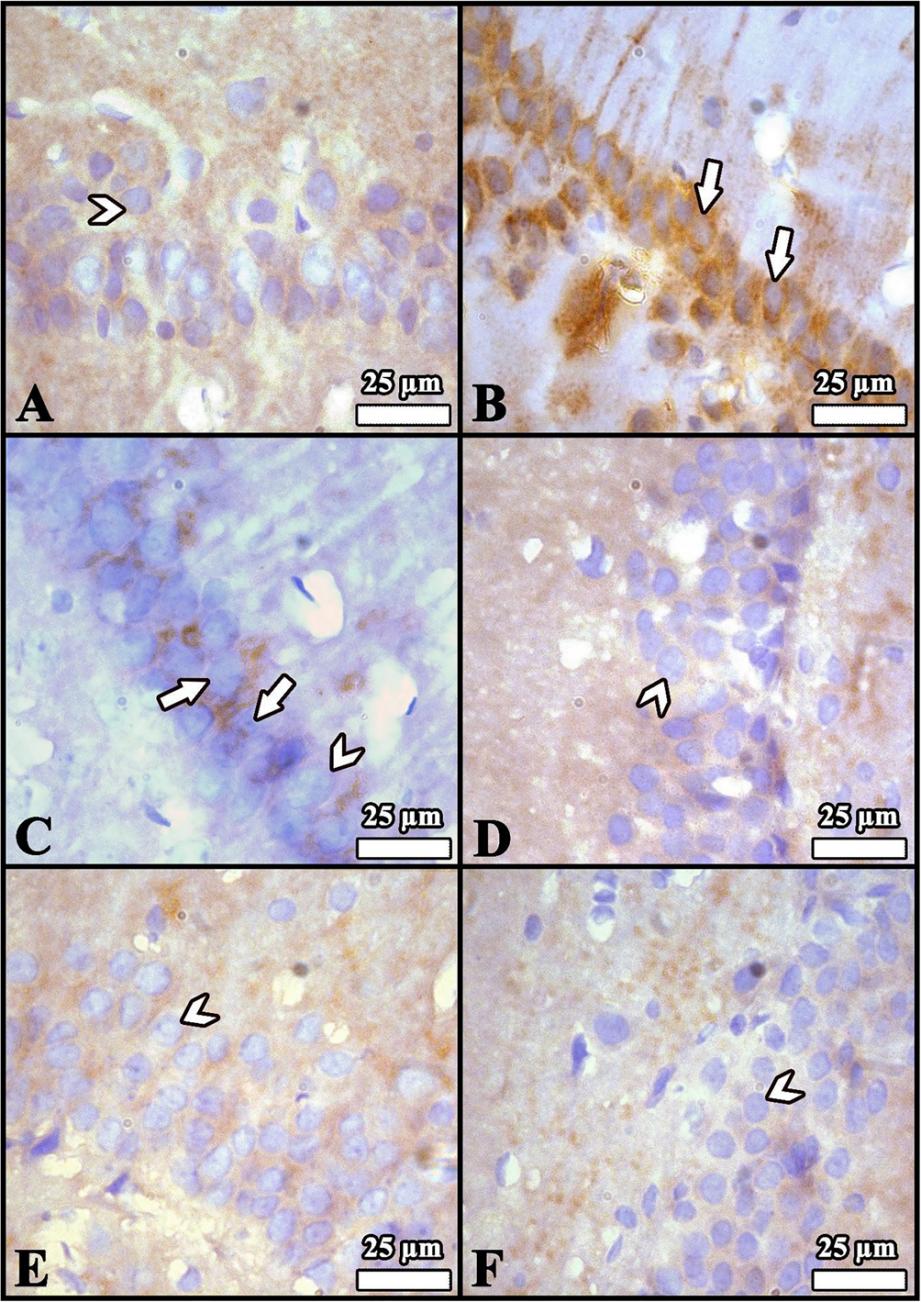
**Light micrographs showing Caspase-3 immunohistochemical reactivity for hippocampal sections from all groups (n: 8).** Cells in the hippocampus were marked with the Caspase-3 antibody, and those which exhibited a positive reaction were brown in colour. (A) Control group; (B) Diabetes mellitus group; (C) Diabetes mellitus + Luteolin group; (D) Luteolin group; (E) Citrate buffer group; (F) Dimethyl sulfoxide (solvent) group. In (B), the density of Caspase-3 positive stained cells was remarkable. In (C), it was revealed that the apoptotic effects caused by diabetes were alleviated by Lut treatment. Arrows: Cells positively stained with Caspase-3; Arrowheads: Cells not stained with Caspase-3. Magnification: ×1000.

Apoptosis in neurons was assessed using a Caspase-3 antibody in thin sections of rat hippocampal tissue. IIDI values were calculated for each group of animals and compared (Figure 4). The IIDI value in the DM group was significantly higher than in all other groups. Additionally, no statistically significant differences were found between the Cont, Lut, CB, and DMSO groups (*P* > 0.05). Caspase-3 immunoreactivity was indicated by brownish staining in the neuronal cytoplasm (Figure 5). The histopathological and immunohistochemical findings from the study groups were consistent with each other.

### Molecular findings

**Figure 6.**
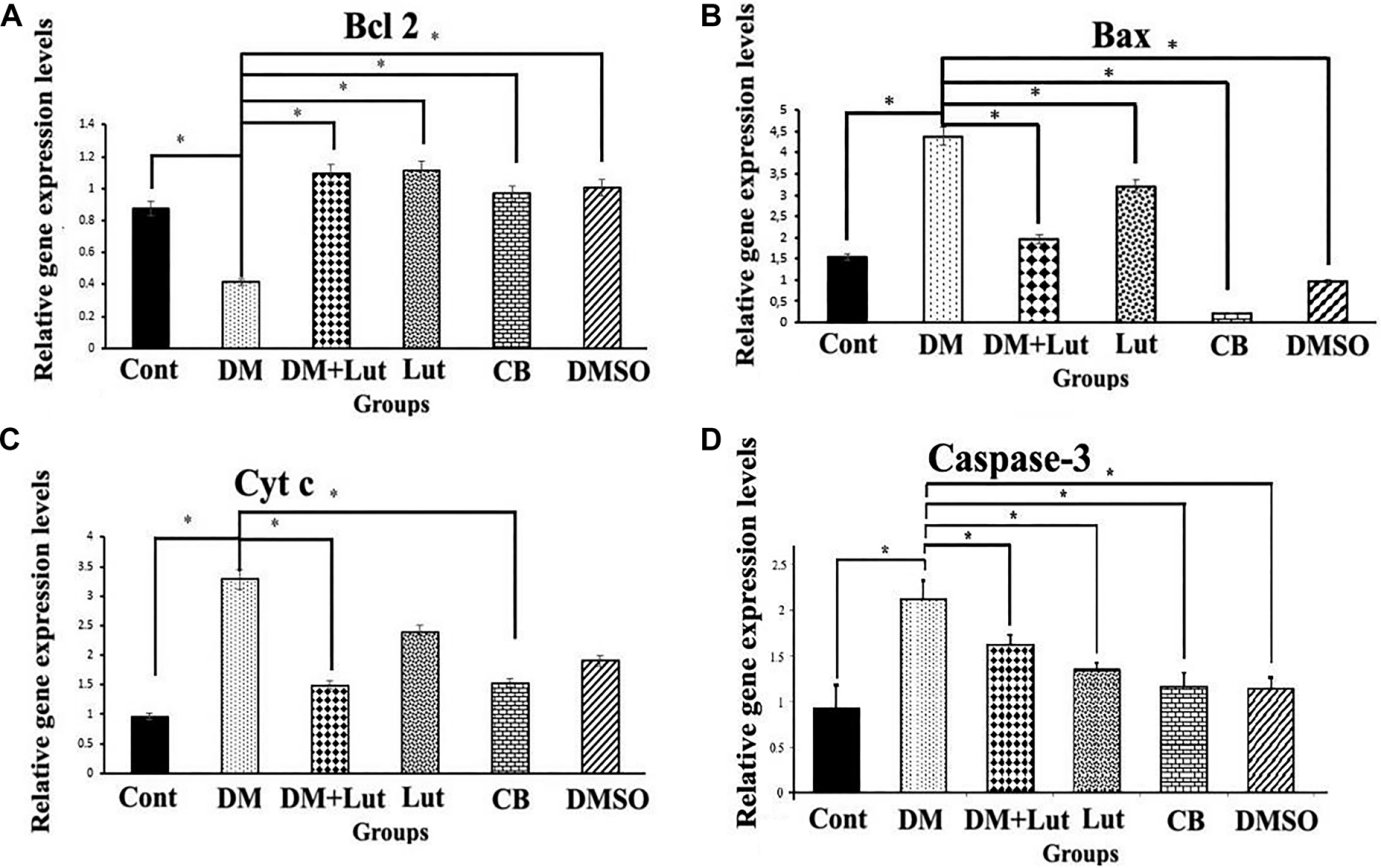
**Bax, Bcl 2, Caspase 3, and Cyt c relative gene expression levels in the hippocampus (*n*: 8).** Differences at the *P* < 0.05 level are indicated by “*.” The data obtained in the relevant graph show that hyperglycemia causes apoptosis in the hippocampus and Lut treatment can prevent apoptosis through molecular pathways. Cont: Control; DM: Diabetes mellitus; Lut: Luteolin; CB: Citrate buffer; DMSO: Dimethyl sulfoxide; Cyt c: Cytochrome c.

**Figure 7.**
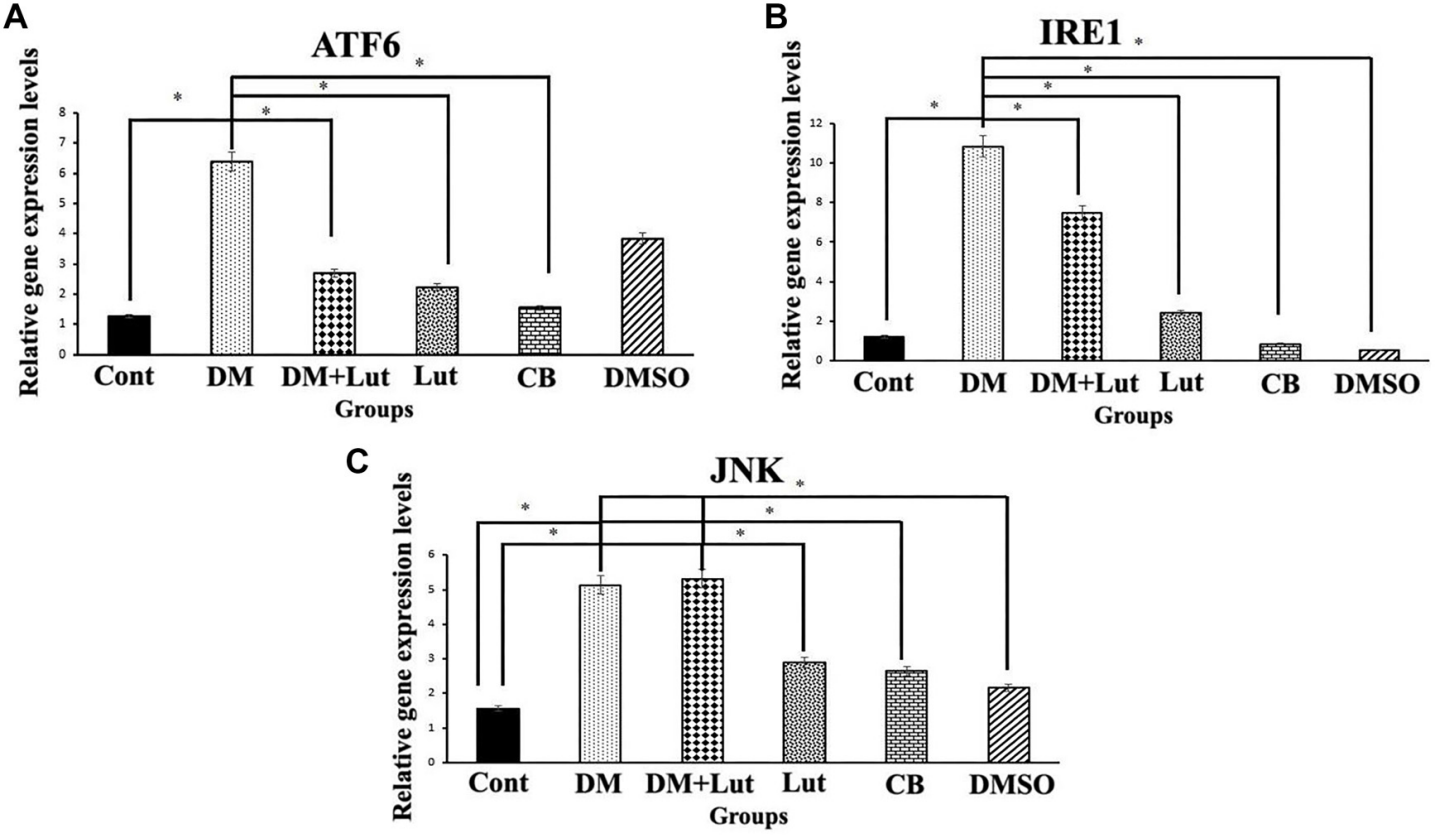
**ATF6, IRE1, and JNK relative gene expression levels in the hippocampus (*n*: 8).** Differences at the *P* < 0.05 level are indicated by “*.” The data obtained in the relevant graph indicate that Lut treatment reduces diabetes-related endoplasmic reticulum stress by acting through ATF6 and IRE1 pathways. Cont: Control; Lut: Luteolin; CB: Citrate buffer; DMSO: Dimethyl sulfoxide; DM: Diabetes mellitus; IRE1: Inositol-requiring enzyme-1; ATF6: Activating transcription factor-6; JNK: c-Jun N-terminal kinase.

Bax, Bcl-2, Caspase-3, and Cyt c expression levels were examined as markers of apoptosis, while ATF6, IRE1, and JNK expression levels were assessed to evaluate ER stress. All genes were analyzed individually in each group, with expression levels normalized to the housekeeping gene GAPDH. Bcl-2 expression was significantly lower in the DM group compared to the Cont, CB, DMSO, DM+Lut, and Lut groups (*P* < 0.05). Conversely, Bax expression was significantly higher in the DM group than in the Cont, CB, DMSO, DM+Lut, and Lut groups (*P* < 0.05). Caspase-3 expression also showed a significant increase in the DM group compared to the Cont, DM+Lut, CB, Lut, and DMSO groups (*P* < 0.05). Additionally, Cyt c expression was significantly elevated in the DM group relative to the Cont, DM+Lut, and CB groups. In the Lut group, Cyt c expression was significantly higher in the CB group than in the Cont group (*P* < 0.05) (Figure 6). Regarding ER stress, ATF6 expression was significantly higher in the DM group compared to the DM+Lut, Cont, Lut, and CB groups (*P* < 0.05). IRE1 expression was also significantly increased in the DM group compared to the Cont, DM+Lut, CB, DMSO, and Lut groups (*P* < 0.05). Lastly, JNK expression was significantly higher in both the DM and DM+Lut groups compared to the Cont, DMSO, and Lut groups (*P* < 0.05) (Figure 7).

### Biochemical findings

**Figure 8.**
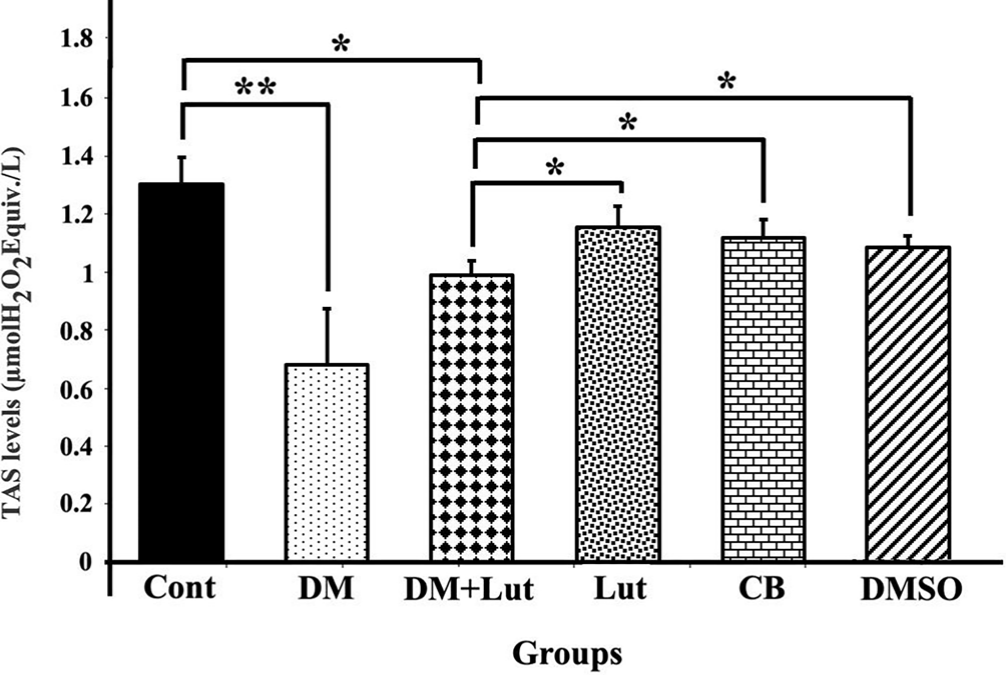
**TAS levels in all the study groups (*n*: 8).** Differences at the *P* < 0.05 level are indicated by “*” and differences at the *P* < 0.01 level by “**.” TAS levels in hippocampal tissue were significantly lower in the DM group than in the Cont, Lut, CB, and DMSO groups (*P* < 0.01). The increase in TAS values in the DM+Lut group was not significant compared with the DM group (*P* > 0.05). Cont: Control; Lut: Luteolin; CB: Citrate buffer; DMSO: Dimethyl sulfoxide; DM: Diabetes mellitus; TAS: Total antioxidant status.

**Figure 9.**
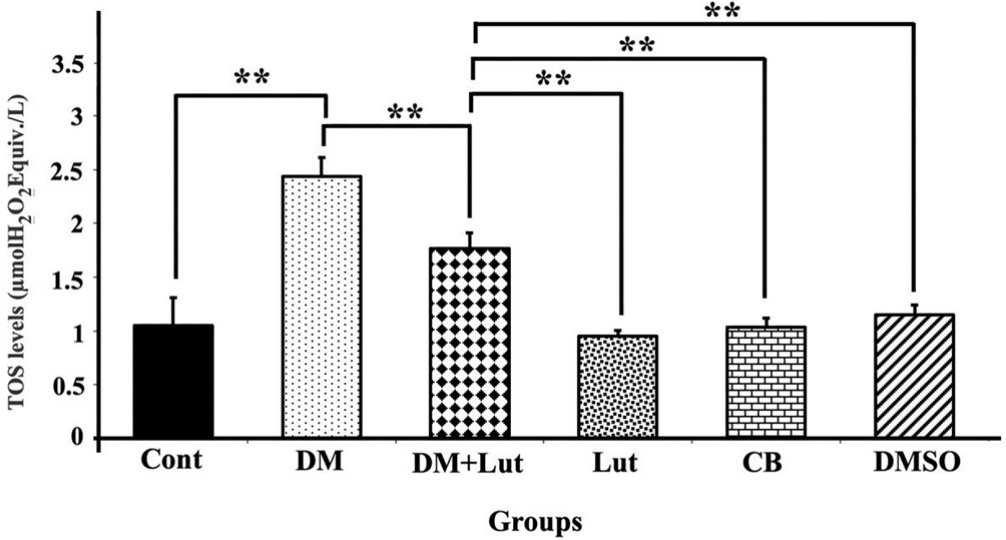
**TOS levels in all the study groups (*n*: 8).** Differences at the *P* < 0.01 level are indicated by “**.” TOS levels in hippocampal tissue were significantly higher in the DM group than in the Cont, Lut, CB, and DMSO groups (*P* < 0.01). Besides the decrease in TOS values in the DM group following the administration of Lut was statistically significant (*P* < 0.01). Cont: Control; Lut: Luteolin; CB: Citrate buffer; DMSO: Dimethyl sulfoxide; DM: Diabetes mellitus; TOS: Total oxidant status.

**Figure 10.**
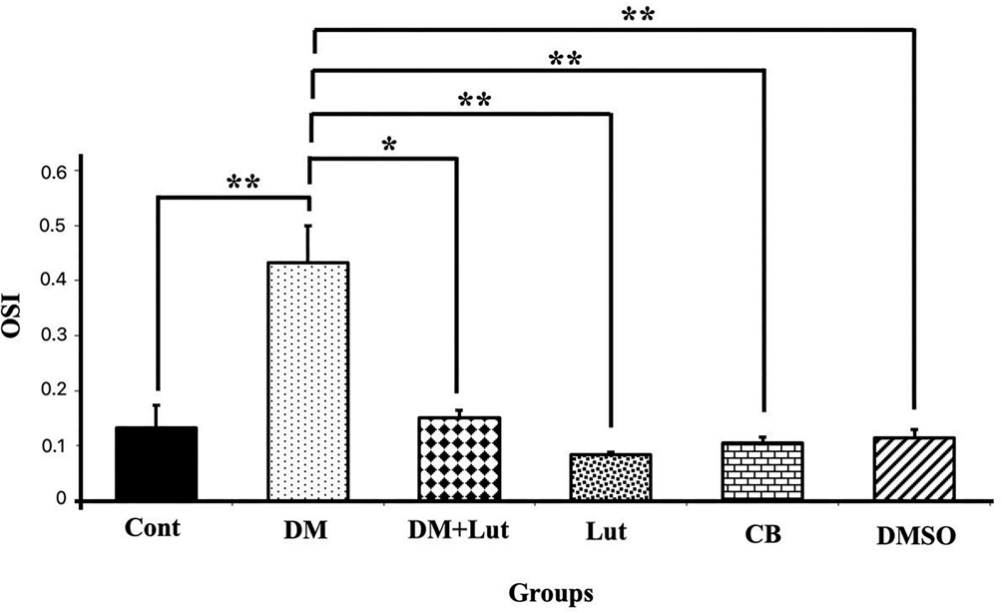
**OSI levels in all the study groups (*n*: 8).** Differences at the *P* < 0.05 level are indicated by “*” and differences at the *P* < 0.01 level by “**.” OSI levels in hippocampal tissue were significantly higher in the DM group than in the Lut, Cont, CB, DMSO (*P* < 0.01) and DM+Lut (*P* < 0.05) groups. No statistical difference was observed between the Cont, CB, DMSO, and Lut groups (*P* > 0.05). Consequently, when the OSI values obtained in the graph were examined, it was seen that the oxidative stress caused by diabetes was alleviated by Lut treatment. Cont: Control; Lut: Luteolin; CB: Citrate buffer; DMSO: Dimethyl sulfoxide; DM: Diabetes mellitus; OSI: Oxidative stress index.

Serum samples from all groups were analyzed for oxidative stress index (OSI) values, calculated by measuring TAS and TOS, to assess oxidative stress (Figures 8–10). TAS levels in hippocampal tissue were significantly lower in the DM group compared to the Cont, Lut, CB, and DMSO groups (*P* < 0.01), while TOS levels were significantly higher (*P* < 0.01). Furthermore, although the administration of Lut to the DM group resulted in a statistically significant decrease in TOS levels (*P* < 0.01), the increase in TAS levels in the DM+Lut group was not statistically significant (*P* > 0.05). Additionally, the OSI value was significantly higher in the DM group than in the other groups. When comparing the DM and DM+Lut groups, this increase was reversed, showing a statistically significant decrease in OSI (*P* < 0.05).

## Discussion

The prevalence of DM is increasing rapidly, and the average age of affected individuals is gradually decreasing. This rise signals serious future health challenges and significant increases in healthcare expenditures. The symptoms and complications associated with diabetes represent a major concern. Oral hypoglycemic agents are commonly used for treatment, though new drugs continue to be investigated due to the lack of a definitive cure and the adverse effects of current therapies. Therefore, extensive research into anti-diabetic compounds is critical to alleviate diabetes-related complications. Hyperglycemia leads to the formation of free radicals through protein glycosylation and glucose oxidation. As a result, antioxidant activity decreases and oxidative stress ensues [32]. The brain is particularly vulnerable to ROS due to its high metabolic rate, reduced regenerative capacity, and elevated concentrations of polyunsaturated fatty acids [33]. Studies have demonstrated increased oxidative stress in diabetes, which contributes to brain damage [4, 34, 35]. Thus, DM is not only a chronic metabolic disorder but also a condition marked by elevated oxidative stress. Excess free radicals in diabetes interact with lipids, proteins, and nucleic acids, leading to compromised membrane integrity, altered protein structure and function, and genetic mutations. Such damage in the CNS may predispose individuals to neurodegenerative diseases, such as Alzheimer’s and Parkinson’s by disrupting protein structures and exocytotic pathways under conditions of chronic hyperglycemia [36]. Cognitive impairment can arise when the hippocampus—a region rich in insulin receptors—is affected by diabetes-induced inflammation and apoptosis [5, 37, 38]. Although the exact mechanisms remain unclear, changes in hippocampal neurogenesis are thought to contribute. Research has shown that DM leads to insulin resistance in the brain and a reduction in hippocampal neurons [37, 39]. The body possesses enzymatic and non-enzymatic antioxidant defense systems to combat the harmful effects of these radicals [32]. However, when the balance shifts in favor of free radicals, antioxidant compounds have been shown to reduce oxidative stress and neural damage by inhibiting radical activity [40, 41]. The ER is a crucial organelle responsible for protein folding. Pathological conditions—including protein misfolding, inflammation, oxidative stress, and hypoxia—can impair ER function [42]. Disruptions during protein maturation result in protein accumulation within the ER, triggering ER stress. This stress activates specific response pathways implicated in numerous diseases. Studies have established links between ER stress and conditions, such as diabetes, Alzheimer’s disease, Parkinson’s disease, ischemia, atherosclerosis, prion diseases, cancer, autoimmune disorders, and cardiovascular disease [43, 44]. ER stress also impairs pancreatic β-cell function and promotes apoptosis, directly contributing to the pathogenesis of DM [45]. Managing DM involves dietary modifications, lifestyle changes, oral hypoglycemics, exogenous insulin, and herbal remedies. While synthetic medications are often associated with adverse side effects, natural substances—particularly plants and herbs—offer glucose-lowering effects with fewer side effects, due to bioactive phytochemicals, such as flavonoids, saponins, alkaloids, tannins, glycosides, and terpenes [46]. Lut is a thermostable flavonoid with antioxidant, antimicrobial, anti-inflammatory, cardioprotective, antidiabetic, neuroprotective, and anti-allergic properties [47]. Lut has been recognized for its capacity to stimulate multiple biological processes, supporting its potential as an effective antidiabetic agent [48]. In a study by Ren et al. [49], Lut was administered to rats with diabetic encephalopathy, and a significant reduction in hippocampal neurons was observed in the diabetic group compared with Cont, Lut, and DMSO groups (*P* < 0.01). Following 15 days of Lut treatment, neuron numbers returned to normal levels. Similarly, in a 22-day study by Abbas et al. [50] involving mice injected with STZ to model Alzheimer’s disease, hippocampal gliosis and neuronal degeneration were observed in the STZ group, while Lut treatment exerted protective effects and improved neuronal survival. Additionally, Kahksha et al. [47] reported that Lut exerts antidiabetic effects by mitigating hyperlipidemia, oxidative stress, and pro-inflammatory conditions in diabetic models, suggesting its promise as a therapeutic agent for DM.

Lut exhibits a range of pharmacological properties that suggest its potential for therapeutic use, including antioxidant, anti-inflammatory, antidiabetic, and possible anticancer activities. However, its safety profile has not been fully established. While numerous studies have highlighted its beneficial effects, a comprehensive assessment of its safety and toxicity is crucial for clinical application. Understanding the potential adverse effects and toxicological considerations associated with Lut is essential for its safe and effective therapeutic use [51]. Orji et al. [52] investigated the acute toxicity of Lut and its effects on various hematological and hepatic function parameters in animal models. Their study found that the LD50 (lethal dose for 50% of the population) of pure Lut exceeded 5000 mg/kg, indicating that it is not acutely toxic. Nevertheless, caution is advised with prolonged use, particularly at high doses, as it may not be entirely without risk. In another study, Wang et al. [53] reported that an eight-week Lut treatment helped prevent diabetes-induced morphological damage to the kidney and improved the redox balance within renal tissue. These findings suggest that long-term regulation of antioxidant levels through Lut supplementation may help prevent the progression of diabetes and its related complications such as nephropathy. Despite these promising results, further research into the molecular mechanisms of Lut is necessary. Such studies will provide insights that can guide the development of new Lut-based therapies and broaden its application in various fields. Histopathological analysis in the present study revealed a higher number of degenerated pyramidal neurons in the diabetes group compared to the other groups. Sugimoto et al. [31] reported that damaged neurons typically exhibit three main features: irregular cellular outlines, increased chromatin density in the nucleus, and greater chromatin density in the cytoplasm. Consistent with these findings, our diabetes group showed poorly defined cell boundaries and a higher prevalence of neurons with thin, dark-stained cytoplasm relative to the other groups. El-Adli et al. [54] similarly observed numerous shrunken, dark-stained pyramidal cells in hippocampal sections from diabetic animals. In line with this, Amin et al. [55] noted multiple small, dark pyramidal neurons, disorganized layers, and vacuolization in the hippocampus of diabetic rats. In contrast, our study found that the DM+Lut group displayed more neurons with normal morphology compared to the DM group, suggesting a neuroprotective effect of Lut against diabetes-induced hippocampal damage. No significant histopathological differences were observed between the Cont, DMSO, and Lut-only groups, supporting the conclusion that Lut mitigates neuronal degeneration associated with diabetes. Similarly, Xiong et al. [56] demonstrated that Lut alleviated pathological changes associated with diabetic nephropathy. GFAP, an intermediate filament protein expressed in astrocytes, serves as a marker for astrocyte activation in the CNS [30]. Reactive astrogliosis, which is characterized by increased GFAP expression, occurs in response to CNS injury or oxidative stress [57]. A notable finding of the present study was the elevated GFAP expression throughout the hippocampus in the DM group, indicating enhanced astrocyte activation. Calcium influx (Ca^2+^) has been shown to induce reactive astrogliosis [58], which is part of the early astrocytic response to CNS injury [59]. Interactions between neurons and glial cells are crucial for maintaining Ca^2+^-dependent neuronal activity and synaptic function [60]. In the context of diabetes, disrupted Ca^2+^ homeostasis and impaired signal transmission may lead to neuronal apoptosis. In our study, the DM+Lut group showed reduced GFAP expression compared to the DM group, suggesting that Lut diminishes glial activation and helps prevent reactive gliosis. These results are consistent with findings by Moghaddam et al. [61] and El-Adli et al. [54], who also reported increased astrocyte numbers in diabetic hippocampal regions. Notably, to our knowledge, this is the first study to investigate the effect of Lut on GFAP expression in the diabetic rat hippocampus using immunohistochemistry, highlighting the originality of our research. Hyperglycemia is known to induce oxidative damage, elevate the production of mitochondrial-specific proteins, and activate mitochondrial permeability transition pores. These changes lead to the activation of caspase-3 and caspase-8, two key enzymes involved in apoptosis and cell degeneration [62]. Caspase-3, in particular, is a crucial death protease responsible for cleaving several important cellular proteins during the early stages of apoptosis. It plays a central role in cell lysis and the formation of apoptotic bodies [63]. In this study, caspase-3 immunostaining was performed on hippocampal tissue sections to assess neuronal apoptosis. The results showed significantly higher IIDI values in the DM group compared to the Cont group, indicating increased apoptosis.

These findings are consistent with those of Ünver Sarayaydın et al. [64], who also reported elevated numbers of apoptotic cells in diabetic animals. In contrast, the IIDI value was significantly lower in the DM+Lut group compared to the DM group, suggesting that Lut’s antidiabetic properties may contribute to reducing or preventing diabetic complications by mitigating neuronal apoptosis.

Lut exhibits a range of pharmacological properties that suggest its potential for therapeutic use, including antioxidant, anti-inflammatory, antidiabetic, and possible anticancer activities. However, its safety profile has not been fully established. While numerous studies have highlighted its beneficial effects, a comprehensive assessment of its safety and toxicity is crucial for clinical application. Understanding the potential adverse effects and toxicological considerations associated with Lut is essential for its safe and effective therapeutic use [51]. Orji et al. [52] investigated the acute toxicity of Lut and its effects on various hematological and hepatic function parameters in animal models. Their study found that the LD50 (lethal dose for 50% of the population) of pure Lut exceeded 5000 mg/kg, indicating that it is not acutely toxic. Nevertheless, caution is advised with prolonged use, particularly at high doses, as it may not be entirely without risk. In another study, Wang et al. [53] reported that an 8-week Lut treatment helped prevent diabetes-induced morphological damage to the kidney and improved the redox balance within renal tissue. These findings suggest that long-term regulation of antioxidant levels through Lut supplementation may help prevent the progression of diabetes and its related complications, such as nephropathy. Despite these promising results, further research into the molecular mechanisms of Lut is necessary. Such studies will provide insights that can guide the development of new Lut-based therapies and broaden its application in various fields. Histopathological analysis in the present study revealed a higher number of degenerated pyramidal neurons in the diabetes group compared to the other groups. Sugimoto et al. [31] reported that damaged neurons typically exhibit three main features: irregular cellular outlines, increased chromatin density in the nucleus, and greater chromatin density in the cytoplasm. Consistent with these findings, our diabetes group showed poorly defined cell boundaries and a higher prevalence of neurons with thin, dark-stained cytoplasm relative to the other groups. El-Adli et al. [54] similarly observed numerous shrunken, dark-stained pyramidal cells in hippocampal sections from diabetic animals. In line with this, Amin et al. [55] noted multiple small, dark pyramidal neurons, disorganized layers, and vacuolization in the hippocampus of diabetic rats. In contrast, our study found that the DM+Lut group displayed more neurons with normal morphology compared to the DM group, suggesting a neuroprotective effect of Lut against diabetes-induced hippocampal damage. No significant histopathological differences were observed between the Cont, DMSO, and Lut-only groups, supporting the conclusion that Lut mitigates neuronal degeneration associated with diabetes. Similarly, Xiong et al. [56] demonstrated that Lut alleviated pathological changes associated with diabetic nephropathy. GFAP, an intermediate filament protein expressed in astrocytes, serves as a marker for astrocyte activation in the CNS [30]. Reactive astrogliosis, which is characterized by increased GFAP expression, occurs in response to CNS injury or oxidative stress [57]. A notable finding of the present study was the elevated GFAP expression throughout the hippocampus in the DM group, indicating enhanced astrocyte activation. Calcium influx (Ca^2+^) has been shown to induce reactive astrogliosis [58], which is part of the early astrocytic response to CNS injury [59]. Interactions between neurons and glial cells are crucial for maintaining Ca^2+^-dependent neuronal activity and synaptic function [60]. In the context of diabetes, disrupted Ca^2+^ homeostasis and impaired signal transmission may lead to neuronal apoptosis. In our study, the DM+Lut group showed reduced GFAP expression compared to the DM group, suggesting that Lut diminishes glial activation and helps prevent reactive gliosis. These results are consistent with findings by Moghaddam et al. [61] and El-Adli et al. [54], who also reported increased astrocyte numbers in diabetic hippocampal regions. Notably, to our knowledge, this is the first study to investigate the effect of Lut on GFAP expression in the diabetic rat hippocampus using immunohistochemistry, highlighting the originality of our research. Hyperglycemia is known to induce oxidative damage, elevate the production of mitochondrial-specific proteins, and activate mitochondrial permeability transition pores. These changes lead to the activation of caspase-3 and caspase-8, two key enzymes involved in apoptosis and cell degeneration [62]. Caspase-3, in particular, is a crucial death protease responsible for cleaving several important cellular proteins during the early stages of apoptosis. It plays a central role in cell lysis and the formation of apoptotic bodies [63]. In this study, caspase-3 immunostaining was performed on hippocampal tissuesections to assess neuronal apoptosis. The results showed significantly higher IIDI values in the DM group compared to the Cont group, indicating increased apoptosis. These findings are consistent with those of Ünver Sarayaydın et al. [64], who also reported elevated numbers of apoptotic cells in diabetic animals. In contrast, the IIDI value was significantly lower in the DM+Lut group compared to the DM group, suggesting that Lut’s antidiabetic properties may contribute to reducing or preventing diabetic complications by mitigating neuronal apoptosis.

TAS, TOS, and OSI values were calculated during the biochemical examinations. OSI, an oxidative stress marker, represents the ratio of total oxidants to antioxidants in serum. While TAS is a crucial factor in preventing diabetes-induced oxidative stress, TOS may reflect the level of free radicals that contribute to diabetes-related cellular degradation [65]. Dağsuyu et al. [66] reported significantly increased ROS and TOS levels and significantly decreased TAS values in the brain and cerebellar tissues of diabetic rats compared to a Cont group. These effects were reversed with metformin treatment. The results of the present study showed that Lut exhibited similar effects to metformin, improving TOS values through its antioxidant properties. However, the increase in TAS in the DM+Lut group was not statistically significant. This lack of significant change suggests that the existing pathology might not be severe enough to alter overall antioxidant capacity. Nonetheless, the data indicate that more detailed studies are needed to evaluate the full extent of Lut’s effects on diabetes. Additionally, since these analyses were performed on peripheral blood, it remains unclear how accurately they reflect tissue-level damage or whether specific alterations occur within affected tissues—issues that warrant further investigation. Ahmad et al. [67] investigated the effects of Lut on Aβ 1–42-induced neuronal apoptosis in mouse brains. They found increased expression of pro- apoptotic markers, such as Bax, Caspase-3, and Cox-2, alongside decreased expression of anti- apoptotic markers. Immunofluorescence staining showed that Caspase-3 expression specifically increased in the frontal cortex and dentate gyrus regions of the hippocampus in the Aβ 1–42-injected group and significantly decreased following Lut treatment. In another study, Lut administered to protect against H_2_O_2_-induced oxidative damage in PC12 neuronal cells led to decreased expression of pro-apoptotic proteins Bax, cleaved/precursor (c/p) Caspase-9, and c/p Caspase-3, while increasing levels of the anti-apoptotic protein Bcl-2. These findings demonstrated Lut’s ability to protect PC12 cells from oxidative stress by inhibiting the apoptotic pathway [68]. Other researchers have shown that Lut neutralizes free radicals induced by STZ, thereby protecting cells from apoptosis by modulating pro-apoptotic gene expression. Western blot analysis of diabetic brain tissue revealed increased expression of Caspase-3, Bax, and Cyt c in the diabetic group. However, this expression was downregulated in the group treated with 10 mg/kg Lut for 15 days, supporting the present study’s findings that Lut inhibits apoptosis via the mitochondrial pathway [49]. Similarly, another study reported that Lut treatment increased Bcl-2 expression while reducing levels of pro-apoptotic proteins, such as Bax, Cyt c, cleaved Caspase-3, and cleaved Caspase-9 in a model of Alzheimer’s disease-induced neuronal apoptosis [69]. Yu et al. [70] also found a higher Bcl-2/Bax ratio in Lut-treated groups compared to Aβ-injected groups, suggesting that Lut supports cellular homeostasis, regulates Bcl-2 family proteins, protects against apoptosis, and reduces neurodegeneration. Qiao et al. [71], using a cerebral ischemia model, found that Lut administration significantly increased Bcl-2 expression at both the protein and mRNA levels, as confirmed by RT-PCR analysis. Data from the present study indicate that hyperglycemia may induce oxidative stress in neurons, and that Lut treatment mitigates this stress by modulating hyperglycemia-induced degradation via antioxidant mechanisms, ultimately preventing apoptosis through molecular pathways. In this study, expression of UPR signaling pathway transmembrane proteins, such as IRE1 and ATF6—both sensitive to ER stress—was examined, along with downstream pro-apoptotic molecules such as JNK. These markers were evaluated to assess ER stress associated with diabetic neuroinflammation and the effect of Lut treatment. Kou et al. [72] found that Lut reduced IRE1 and GRP78 expression—both indicators of ER stress—in Alzheimer’s disease models, suggesting Lut plays a suppressive role in ER activation and inflammatory signaling pathways in both cellular and animal models. Tana and Nakagawa [21] similarly reported that Lut regulates gene expression in the brain, suppresses ER stress, and reduces inflammation. In the present study, gene expression levels of ATF6 and IRE1 were elevated in the diabetic group but decreased following Lut treatment. However, JNK expression remained high in both the diabetic and Lut-treated groups. These results suggest that Lut mitigates ER stress by acting through the ATF6 and IRE1 pathways. Consequently, Lut may reduce apoptosis and neuronal loss in the hippocampus by targeting components of the UPR pathway affected by diabetes-induced ER stress. In conclusion, the results indicate that diabetes elevates lipid peroxidation and ROS production, activates the IRE1 and ATF6 pathways, and promotes a pro-apoptotic state leading to hippocampal neuronal damage. Lut treatment reduced oxidative stress and enhanced ER-associated degradation through modulation of IRE1 and ATF6, and was associated with observed neuronal protection. These findings suggest that Lut may prevent diabetes-induced hippocampal damage by attenuating both oxidative and ER stress.

In light of this information, several medicines are used to manage diabetes; however, prolonged use of synthetic drugs can lead to adverse effects in diabetic patients, including episodes of hypoglycemia and gastrointestinal issues, such as vomiting, nausea, and diarrhea [73]. Herbal medicines or their extracts have long been used to treat various diseases, as they are generally perceived as less harmful and associated with fewer side effects compared to synthetic alternatives [53]. Lut has demonstrated strong anti-diabetic properties, which have been investigated globally. Research using cell lines and animal models has substantiated its efficacy as an anti-diabetic agent [48]. The present study comprehensively demonstrates the therapeutic effects of Lut on diabetes through molecular, histological, and biochemical approaches, revealing promising results. Although preclinical studies indicate the potential therapeutic benefits of Lut in diabetes, translating these findings into clinical practice is essential. Clinical investigations are crucial for evaluating the efficacy, safety, and therapeutic potential of Lut in humans. Despite the extensive preclinical evidence supporting its anti-inflammatory, antioxidant, anti-diabetic, and anti-cancer properties, the clinical applicability of Lut requires further examination to confirm its benefits and determine effective dosing regimens. In conclusion, the pharmacological properties of Lut underscore its significance, necessitating continued research and clinical evaluation to fully uncover its therapeutic potential for human health.

In this study, the effects of Lut on apoptosis, oxidative stress, and ER stress pathways were comprehensively evaluated at both the molecular and histological levels. However, several important limitations should be noted. The neuroprotective effects of Lut were primarily investigated through mechanisms related to cell death and stress pathways. Other potential neuroprotective mechanisms, such as synaptic plasticity and neurogenesis, could not be assessed. Future studies will examine synaptic plasticity markers—including BDNF, Synapsin-1, and PSD-95—to provide a more comprehensive understanding of Lut’s neuroprotective effects. Another limitation of this study was the inability to verify results at the protein level. Western blot and/or immunofluorescence analyses were not feasible due to limited resources. We intend to use these techniques in future studies to quantitatively assess proteins, such as p-JNK, CHOP, and GRP78. Although the neuroprotective effects of Lut were supported by molecular and histological findings, behavioral assessments, such as the Morris water maze and Y-maze were not included to evaluate cognitive function. Since the main objective of this study was to explore the effects of Lut on apoptosis, oxidative stress, and ER stress pathways, behavioral tests were excluded from the study design. Nonetheless, evaluating learning, memory, and anxiety-related behaviors using cognitive function tests is crucial for directly linking hippocampal protection to functional recovery. Therefore, it is recommended that future studies include behavioral assessments to better verify Lut’s effects on cognitive function. In this context, we believe that comprehensive future studies will significantly contribute to the scientific literature by addressing these limitations and further elucidating the mechanisms of action of Lut.

## Conclusion

Finally, given its high incidence, secondary complications, and prolonged treatment duration, DM imposes a significant burden on healthcare systems worldwide. Therefore, it is crucial to conduct research aimed at mitigating the complications associated with diabetes. In this context, the present study demonstrated that Lut treatment may protect against STZ-induced diabetic hippocampal damage by reducing oxidative stress and ER stress in hippocampal tissue. Additionally, Lut improved acute hyperglycemia-induced hippocampal injury by inhibiting pro-apoptotic pathway proteins. This study is among the first to reveal the potential protective effects of Lut against diabetes-induced hippocampal damage and offers new insights into its neuroprotective and therapeutic mechanisms. We hope our findings will contribute to the development of novel therapeutic strategies for diabetes-related hippocampal damage and help reduce hippocampal toxicity in diabetic individuals. However, ER stress and its related signaling pathways are known to play a role in the pathogenesis of various disease groups. Future studies should investigate how diabetes-associated changes in ER stress responses—particularly in pathways that either hinder or promote disease progression—contribute to the condition. Continued research into oxidative stress and ER stress will provide essential insights to guide and improve future clinical studies in this field.
